# Mouse genetic background influences whether *Hras^G12V^* expression plus *Cdkn2a* knockdown causes angiosarcoma or undifferentiated pleomorphic sarcoma

**DOI:** 10.18632/oncotarget.24831

**Published:** 2018-04-13

**Authors:** Laura P. Brandt, Joachim Albers, Tomas Hejhal, Svende Pfundstein, Ana Filipa Gonçalves, Antonella Catalano, Peter J. Wild, Ian J. Frew

**Affiliations:** ^1^ Institute of Physiology, University of Zurich, Zurich, Switzerland; ^2^ Zurich Center for Integrative Human Physiology, University of Zurich, Zurich, Switzerland; ^3^ Zurich Integrative Rodent Physiology, University of Zurich, Zurich, Switzerland; ^4^ Department of Pathology and Molecular Pathology, University Hospital Zurich, Zurich, Switzerland; ^5^ BIOSS Centre for Biological Signaling Studies, University of Freiburg, Freiburg, Germany; ^6^ Department of Hematology, Oncology and Stem Cell Transplantation, Faculty of Medicine, Medical Center, University of Freiburg, Freiburg, Germany

**Keywords:** angiosarcoma, undifferentiated pleomorphic sarcoma, MuLE lentivirus, H-Ras, mouse model

## Abstract

Soft tissue sarcomas are rare mesenchymal tumours accounting for 1% of adult malignancies and are fatal in approximately one third of patients. Two of the most aggressive and lethal forms of soft tissue sarcomas are angiosarcomas and undifferentiated pleomorphic sarcomas (UPS). To examine sarcoma-relevant molecular pathways, we employed a lentiviral gene regulatory system to attempt to generate *in vivo* models that reflect common molecular alterations of human angiosarcoma and UPS. Mice were intraveneously injected with MuLE lentiviruses expressing combinations of shRNA against *Cdkn2a*, *Trp53*, *Tsc2* and *Pten* with or without expression of *Hras^G12V^*, *PIK3CA^H1047R^* or *Myc*. The systemic injection of an ecotropic lentivirus expressing oncogenic *Hras^G12V^* together with the knockdown of *Cdkn2a* or *Trp53* was sufficient to initiate angiosarcoma and/or UPS development, providing a flexible system to generate autochthonous mouse models of these diseases. Unexpectedly, different mouse strains developed different types of sarcoma in response to identical genetic drivers, implicating genetic background as a contributor to the genesis and spectrum of sarcomas.

## INTRODUCTION

Soft tissue sarcomas are rare mesenchymal malignancies that account for approximately 1% of all cancers. The WHO has defined over 100 different soft tissue sarcoma subtypes named after the tissue that they most closely resemble [1]. Based on molecular characteristics, soft tissue sarcomas can be divided in two broad categories: sarcomas with simple karyotypes, such as chromosomal translocations, and sarcomas with more complex genetic profiles, including *TP53* mutation, *CDKN2A* deletion and *MDM2* amplification [2–4].

Undifferentiated pleomorphic sarcomas (UPS), previously referred to as malignant fibrous histiocytomas (MFH), account for approximately 5% of adult soft tissue sarcomas and represent one of the most common types of high-grade soft tissue sarcoma. Standard treatment options are surgical resection, radiotherapy, and chemotherapy, which in many cases are not curative, highlighting the necessity to develop novel targeted treatments. It is not clear whether UPS represents a group of de-differentiated sarcomas that share a common morphology but which originated from different cell types or if all UPS tumours arise from an as-yet-unidentified common cell of origin [5]. The genetic alterations responsible for the development of UPS are also incompletely understood. *TP53* alterations have been identified in 17% of human UPS [2] and *CDKN2A* loss seems to be an alternative to *TP53* deletion [3]. *HRAS* and *KRAS* mutations have been identified in up to 50% of human UPS tumours [6–8]. Mouse studies have confirmed that the cooperation of oncogenic *Kras* and *Trp53* or *Cdkn2a* deficiency resulted in the development of undifferentiated pleomorphic sarcomas in different tissues [9–12].

Another clinically aggressive subtype of high-grade soft tissue sarcoma is angiosarcoma. These tumours represent rare malignancies of endothelial differentiation that account for approximately 1% of all soft tissue sarcomas. Angiosarcomas show a wide anatomic distribution and arise spontaneously or secondarily to radiation, toxic chemicals (e.g. vinyl chloride) or chronic lymphoedema (Stewart-Treves syndrome). Treatment options are limited and the prognosis is poor [13]. Genetic mutations and amplifications of *VEGF*, *MDM2*, *TP53*, *CDKN2A*, *KRAS* and *MYC* have been described in angiosarcoma patients [14–17]. *MYC* gene amplifications are commonly found in radiation-induced angiosarcomas [18]. A recent publication reported that the majority of genetic alterations were found in the p53 and MAPK pathways. *TP53* was mutated in 35% of the lesions and *CDKN2A* lost in 26%. 53% of angiosarcomas displayed MAPK pathway activation, and harboured genetic activating mutations in *KRAS*, *HRAS*, *NRAS*, *BRAF*, *MAPK1* or inactivating mutations in *NF1* and *PTPRB1* [19, 20]. Several *in vivo* mouse studies showed the involvement of loss of function of the p53 tumour suppressor in angiosarcoma development [21–23]. In addition, the *in vivo* deletion of *Cdkn2a* in mice lead to the development of lesions which recapitulate human angiosarcoma, however, only 30% of the mice displayed angiosarcomas within 100 days [24]. Furthermore, alterations in the PI3K/AKT/mTOR pathway have been identified in a small percentage of patients [19, 25, 26] and deletion of *Tsc1*, a tumour suppressor that negatively regulates the pathway, induced the formation of hemangiosarcomas in mice [27]. Another report showed that the *in vivo* deletion of *Notch1* resulted in the development of hepatic angiosarcomas with a penetrance of 86% at 50 weeks after gene deletion [28], although genetic alterations in the Notch pathway have not been reported in human angiosarcomas. Although these studies have been helpful in uncovering aspects of sarcomagenesis, there is limited understanding of the interactions between cooperating genetic alterations.

In this study we employed a mouse genetic approach using the MuLE lentiviral gene regulatory system [10] to functionally test the contributions of different candidate driver oncogenes and tumour suppressor genes to the formation of angiosarcoma and UPS. Different mouse strains were injected intraveneously with ecotropic MuLE lentiviruses expressing combinations of shRNA against *Cdkn2a*, *Trp53, Tsc2* and *Pten* with or without expression of *Hras^G12V^*, *PIK3CA^H1047R^* or *Myc*. Tumour development was monitored by *in vivo* imaging. We successfully generated new models of angiosarcoma and of UPS based on oncogenic *Hras^G12V^* expression in combination with knockdown of *Cdkn2a* or *Trp53*. Unexpectedly, different mouse strains developed different types of sarcoma in response to identical genetic drivers.

## RESULTS

### Expression of oncogenic *Hras^G12V^* plus knockdown of *Cdkn2a* causes angiosarcoma development in SCID/beige mice

To functionally test the contributions of different candidate driver oncogenes and tumour suppressor genes to the formation of angiosarcoma, we generated a panel of lentiviral vectors based on the MuLE system [10] (Supplementary Figure 1A), to induce genetic alterations that reflect some of the most commonly found alterations of human angiosarcomas. We first utilised these ecotropic MuLE lentiviruses expressing combinations of shRNA or shRNA-miR30 against *Cdkn2a, Trp53, Tsc*2 and *Pten* with or without expression of oncogenic *Hras^G12V^,* oncogenic *PIK3CA^H1047R^* or *Myc* vectors to attempt to generate panels of genetically-engineered angiosarcoma cell lines by infecting a disease-relevant cell type, namely primary murine endothelial cells from the spleen (pMSECs). Western blotting and real time PCR assays of puromycin-selected cultured cells infected with these vectors verified that they effectively induced the desired changes in gene expression (Supplementary Figure 1B–1E). Consistent with an oncogenic activity of these genetic changes, all cell lines, with the exception of *Hras^G12V^* expression alone and *Hras^G12V^* expression plus sh*Trp53*, exhibited increased rates of proliferation (Supplementary Figure 2A). The absence of increased proliferation induced by oncogenic *Hras^G12V^* or oncogenic *Hras^G12V^* plus *Trp53* knockdown is likely to be mediated by the upregulation of p16INK4A protein expression observed in pMSEC cells infected with these vectors (Supplementary Figure 1B) as removal of this putative proliferative barrier by knockdown of *Cdkn2a* increased cellular proliferation (Supplementary Figure 2A).

To further investigate potential transformed cellular behaviour we cultured cells of all genotypes on low-attachment plates, however none of the genetic combinations allowed the proliferation of cells as spheres or masses (data not shown), indicating that they do not have anchorage-independent proliferation capacity. We next asked whether these cellular systems might represent experimentally tractable allograft tumour models by injecting wild type pMSECs or sh*Cdkn2a* plus *Hras^G12V^* pMSECs subcutaneously into SCID/beige immunodeficient mice. Within one month of injection, both the wild type and sh*Cdkn2a* plus *Hras^G12V^* cells formed blood-filled lesions (Supplementary Figure 2B) that were lined with CD31-positive endothelial cells with atypical nuclei growing either as single layers or in papillary projections (Supplementary Figure 2C). The injected cells apparently have the capacity to co-opt or integrate into local blood vessels, resulting in large blood-filled vascular structures. Based on the fact that this phenotype also arose following injection of wild type pMSECs in the absence of oncogene activation or tumour suppressor inactivation, we conclude that this *ex vivo* engineered cellular system does not represent a good allograft model system for angiosarcoma.

**Figure 1:**
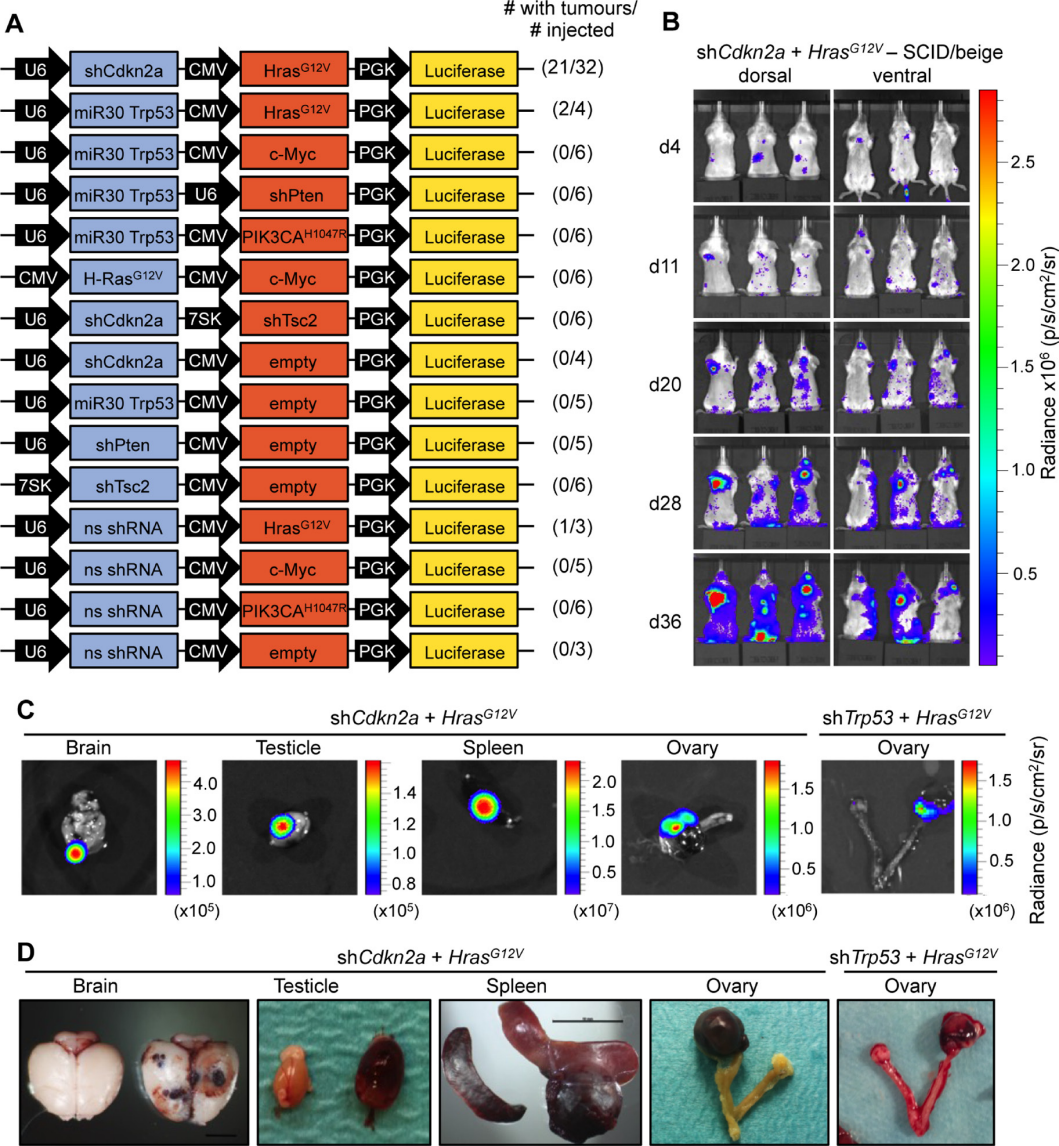
*Hras^G12V^* expression plus knockdown of *Cdkn2a* or *Trp53* causes angiosarcomas in SCID/beige mice (**A**) Schematic of MuLE vectors simultaneously expressing combinations of shRNAs against *Cdkn2a*, *Trp53*, *Pten* and *Tsc2* with or without expression of *Hras^G12V^*, *Myc* or *PIK3CA^H1047R^* and a Luciferase reporter. (**B**) Bioluminescence imaging 4, 11, 20, 28 and 36 days after the injection of MuLE lentiviruses expressing shRNA against *Cdkn2a* together with *Hras^G12V^* into the tail veins of 4-6-week-old SCID/beige mice. Numbers of mice that developed tumours and total number of injected mice are shown for each construct. (**C**) Bioluminescence imaging and (**D**) photographs showing examples of tumour-bearing organs from sh*Cdkn2a* plus *Hras^G12V^* and sh*Trp53* plus *Hras^G12V^* injected animals. Scale bar: 10 mm.

We next sought to assess the tumour forming capacity of the same genetic changes that were tested in the experiments above directly *in vivo*. 4–6-week-old SCID/beige mice were intravenously injected via the tail vein with concentrated ecotropic MuLE vectors that carried an expression element for firefly luciferase (Figure 1A) in order to label infected cells and to trace potential tumour development *in vivo* over time. The intravenous injection of an ecotropic lentivirus expressing oncogenic *Hras^G12V^* together with knockdown of *Cdkn2a* (*n* = 21 of 32 injected mice) or *Trp53* (*n* = 2 of 4 injected mice) in SCID/beige mice induced increases in luciferase signals over 4–8 weeks (Figure 1B and Supplementary Figure 3A). These signals were widely distributed in different organs throughout the body. One of three mice injected with a vector expressing only oncogenic *Hras^G12V^* developed signals in the brain approximately 6 months after injection. None of the other viruses was sufficient to cause any large increases in luciferase signal within 6 months of injection, demonstrating that these combinations of genetic alterations are not oncogenic in this setting.

**Figure 2:**
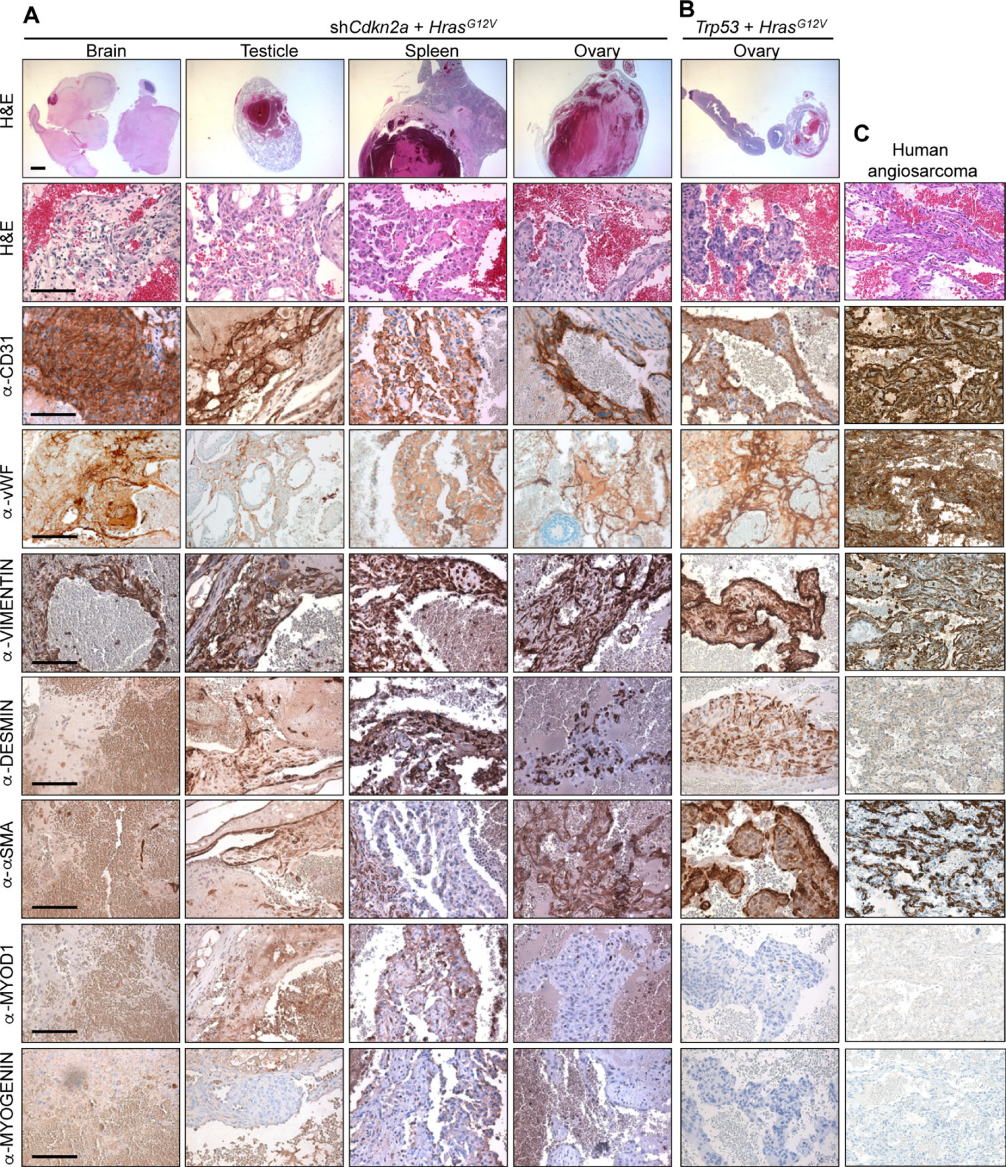
Histological and molecular characterisation of angiosarcomas H&E and immunohistochemical stainings using the indicated antibodies of tumours derived from the systemic injection of (**A**) sh*Cdkn2a* plus *Hras^G12V^* and (**B**) sh*Trp53* plus *Hras^G12V^* viruses. (**C**) Stainings of a human angiosarcoma tumour. Similar staining patterns were observed in two additional cases from different patients. Low magnification scale bars: 1000 μm and high magnification scale bars: 100 μm.

Dissections of mice revealed that the increased luciferase signals corresponded to the presence of bloody-appearing tumours in different organs (Figure 1C and 1D and Supplementary Figure 3). From 32 mice injected with sh*Cdkn2a* plus *Hras^G12V^* MuLE vectors, 21 mice developed a total of 24 tumours in various tissues including testicle (*n* = 9, 38%), brain (*n* = 7, 30%), spleen (*n* = 2, 8%), uterus (*n* = 2, 8%), ovary (*n* = 1, 4%), lung (*n* = 1, 4%), colon (*n* = 1, 4%) and eye (*n* = 1, 4%). Histological analysis of these tumours revealed poorly demarcated malignant neoplasms with hemorrhage and irregular, anastomosing vascular channels. Endothelial lining showed multilayering and intraluminal tufting with nuclear atypia, hyperchromasia, enlargement and irregularity (Supplementary Figure 3A, arrowheads). Mitotic activity was variable and back-to-back vascular channels appeared sieve-like. Atypical cells were either spindled, epitheloid or mixed. There was more solid growth in more poorly differentiated areas. Intraluminal erythrocytes were a common feature as well as large areas filled with red blood cells. Tumour cells exhibited high levels of expression of H-RAS in comparison to surrounding normal tissue, indicating that the MuLE virus is functional *in vivo* (Supplementary Figure 3B). Positive immunoreactivity to antibodies against the endothelial cell marker proteins CD31 and von Willebrand Factor (vWF) confirmed the endothelial differentiation of the tumour cells (Figure 2A). The lesions showed variable staining for VIMENTIN, DESMIN and SMOOTH MUSCLE ACTIN (SMA), ranging from an absence of staining to some tumours showing strong positivity (Figure 2A). None of the tumours exhibited nuclear staining for the skeletal muscle markers MYOD1 and MYOGENIN (Figure 2A). Supplementary Figure 4 shows positive and negative control stainings for the different antibodies employed throughout this study. Tumours that arose in *Trp53* knockdown plus oncogenic *Hras^G12V^* injected mice displayed an identical histological appearance and immunohistochemical staining profile to tumours in *Cdkn2a* knockdown plus oncogenic *Hras^G12V^* injected mice (Figure 2B). In summary, these histological and molecular features are consistent with a diagnosis of angiosarcoma. Indeed, analyses of three human angiosarcomas revealed that they have a simlar histological appearance and exhibit a similar pattern of immunoreactivity to the mouse tumours (Figure 2C). We conclude that we have developed a rapid autochthonous mouse model of angiosarcoma that is trackable via live animal imaging and that reflects the frequent genetic alterations that arise in human angiosarcoma tumours.

### Different immunocompetent mouse strains display different types of soft tissue sarcomas in response to expression of oncogenic *Hras^G12V^* plus loss of *Cdkn2a* function

To more accurately model the complexities of tumour development in humans it would be desirable to have tumour models that arise in immunocompetent mice. We therefore investigated whether similar tumours arose in response to intravenous injection of *shCdkn2a* plus *Hras^G12V^* MuLE vectors in the Fox Chase CB17, 129/Sv and C57BL/6 mouse strains.

Fox Chase CB17 mice carry the immunoglobulin heavy chain allele from C57BL/Ka mice on a BALB/c background. They serve as an ideal control for SCID/beige mice as they represent the identical genetic background but have a normal immune system. Within 4 weeks, 7 of 8 (88%) Fox Chase CB17 mice injected intraveneously with sh*Cdkn2a* plus *Hras^G12V^* MuLE vectors developed angiosarcomas with comparable growth kinetics and histology to those that arose in SCID/beige mice (Supplementary Figure 5A). The anatomic distribution of the tumours in Fox Chase CB17 mice was similar to the tumour distribution seen in SCID/beige mice and included brain (*n* = 4, 50%), testicle (*n* = 3, 38%) and spleen (*n* = 1, 12%) (Supplementary Figure 5B). The tumours presented as bloody lesions and stained positively for CD31 and vWF by immunohistochemistry. Like the tumours in SCID/beige mice, these tumours also showed variable staining for VIMENTIN, DESMIN and SMA as well as an absence of staining for MYOD1 and MYOGENIN (Supplementary Figure 5C). These experiments demonstrate that a competent immune system does not affect tumour formation and provide a new autochthonous angiosarcoma model in an immunocompetent background.

**Figure 3:**
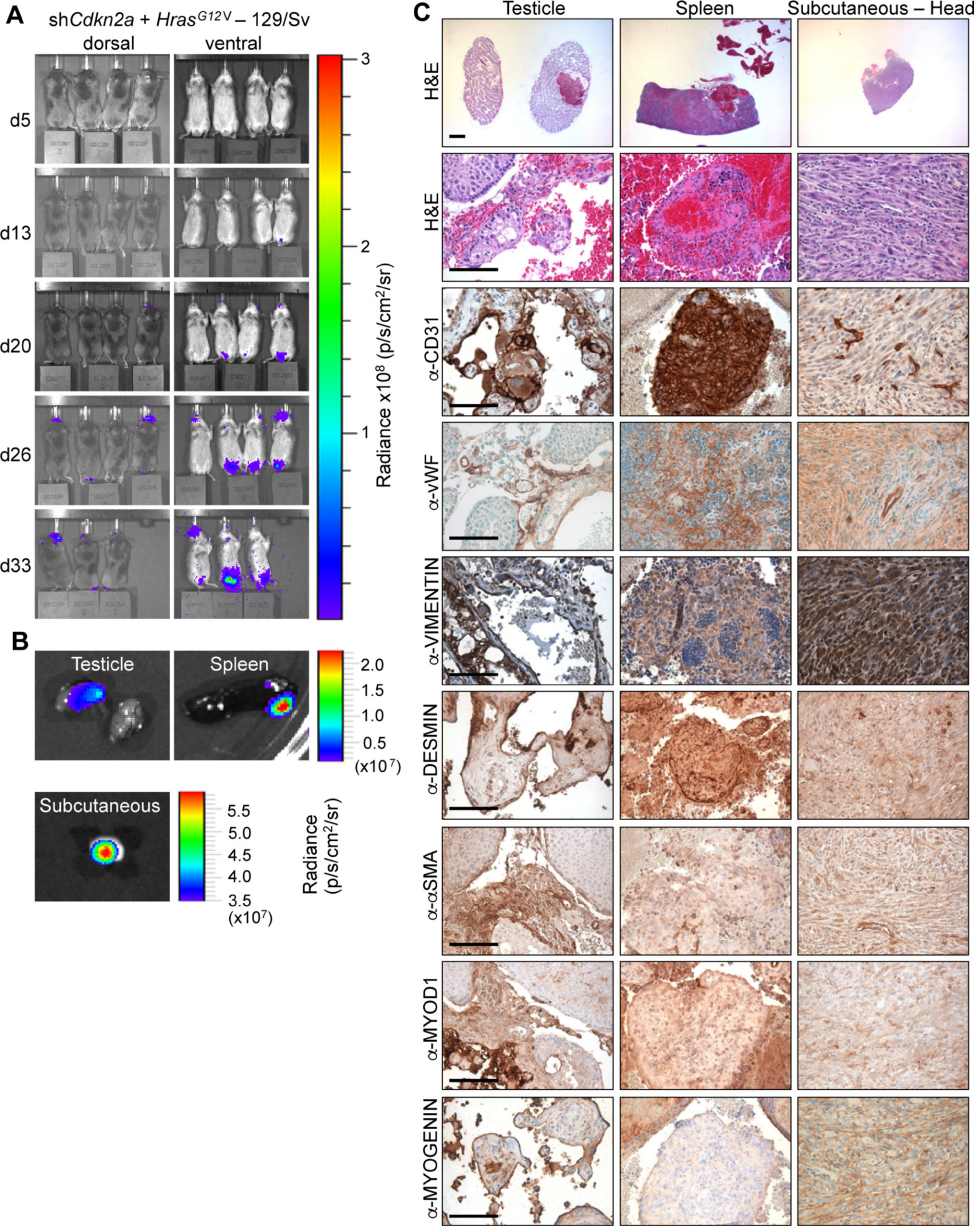
*Hras^G12V^* expression plus knockdown of *Cdkn2a* causes angiosarcomas and undifferentiated pleomorphic sarcomas in 129Sv mice (**A**) Bioluminescence imaging 5, 13, 20, 26 and 33 days after the injection of MuLE lentiviruses expressing shRNA against *Cdkn2a* together with *Hras^G12V^* into the tail veins of 4-6-week-old 129/Sv mice. (**B**) Bioluminescence imaging showing examples of tumour-bearing organs.. (**C**) H&E and immunohistochemical stainings using the indicated antibodies. Low magnification scale bars: 1000 μm and high magnification scale bars: 100 μm.

**Figure 4:**
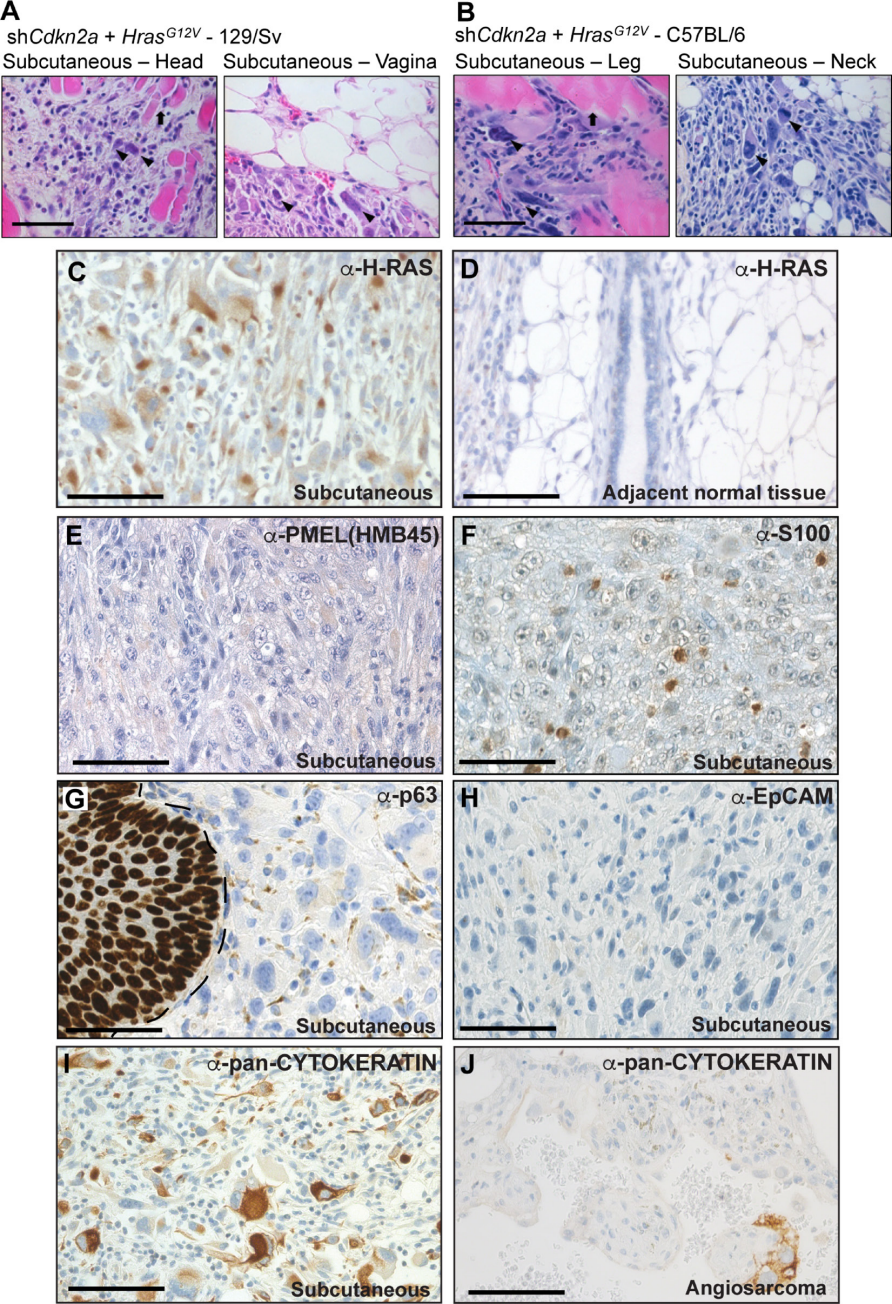
High grade, locally invasive undifferentiated pleomorphic sarcomas form in 129/Sv and C57BL/6 mice (**A**, **B**) High magnification images of H&E stained tumour sections derived from the intraveneous injection of sh*Cdkn2a* plus *H*-*Ras^G12V^* viruses in (A) 129/Sv mice and (B) C57BL/6 mice. Arrowheads indicate atypical nuclei and arrows indicate entrapped muscle fibres. Images depict tumour invasion into the surrounding muscle or fat tissue. (**C**, **D**) Immunohistochemical staining using an anti-H-RAS antibody showing high H-RAS expression in a subcutaneous tumour (C) but absence of staining in adjacent normal tissue (D). (**E**–**I**) Representative immunohistochemical staining patterns for the indicated antibodies in subcutaneous tumours. In (G) the margin between normal skin (p63 nuclear positive) and the tumour is shown by a dotted line. (**J**) Occasional cells in angiosarcoma tumours from SCID/beige mice stain positively for pan-CYTOKERATIN. Scale bars: 100 μm.

To investigate sarcoma formation in mouse backgrounds that are more commonly utilised for biomedical research we next employed 129/Sv mice. Intraveneous injections of sh*Cdkn2a* plus *Hras^G12V^* MuLE viruses in 129/Sv mice (*n* = 8) caused a strong luciferase signal increase and the development of multiple tumours with 100% penetrance within 4 weeks (Figure 3A and 3B, Supplementary Figure 6A). Bloody-appearing tumours were observed in testicles of 75% of male mice (*n* = 3) and in uteri (*n* = 2) and ovaries (*n* = 2) of 50% of female mice. 25% of mice carried lesions in the spleen (*n* = 2), and 13% in lung (*n* = 1) and brain (*n* = 1), similar to results in SCID/beige mice. However, all female mice (*n* = 4) additionally developed subcutaneous tumours (*n* = 5) that were located in the head and neck region and close to the subcutis of the vulva. These tumours were solid and white in appearance (Figure 3C, last column). Neither the sub-cutaneous location, nor the gross morphological appearance, were ever seen in tumours in SCID/beige or Fox Chase CB17 mice. While all of the bloody-appearing tumours exhibited an identical histological appearance and pattern of immunoreactivity similar to tumours that arose in SCID/beige mice (Figure 3C, Supplementary Figure 6B), classifying them as angiosarcomas, the subcutaneous tumours exhibited a completely different histology and immunohistochemical staining profile. These tumours contained cells with rhabdoid features; i.e., large polygonal cells with gigantic bizarre nuclei (Figure 4A; arrowheads), abundant, deeply eosinophilic cytoplasm in a tadpole- or racquet shape and growing in a storiform pattern. Nuclei displayed high mitotic indices, irregular nuclear membranes, and eosinophilic cytoplasmic inclusions. These tumors showed necrotic regions, acute inflammatory responses and were highly invasive, infiltrating surrounding tissues including muscle and fat (Figure 4A, arrows). The absence of apparent features of any definable cell lineage is suggestive of a diagnosis of undifferentiated pleomorphic sarcoma (UPS). Indeed, while these tumours showed high levels of H-RAS expression in comparison to adjacent normal tissue (Figure 4C, 4D) and were immunoreactive for the common mesenchymal marker VIMENTIN, the tumour cells did not stain for lineage markers including CD31, vWF, DESMIN, SMA, MYOD1 or MYOGENIN (Figure 3C, last column). Given the subcutaneous location of these tumours, we further investigated whether the tumours might potentially represent sarcomatoid variants of malignancies derived from a skin cell. Tumours in 129/Sv mice stained negatively for the melanoma marker protein PMEL (the HMB45 antigen) (Figure 4E). Melanomas, as well as other types of neural lineage-derived tumours are typically diffusely positive for S100 and PMEL. The majority of tumour cells in tumours in 129/Sv mice were negative for S100 but some scattered cells within the tumour displayed positive immunoreactivity for S100 (Figure 4F). Since macrophages also stain positively for S100, it is likely that this staining is due to the presence of inflammatory cells. Tumour cells in human sarcomatoid squamous cell carcinomas typically exhibit nuclear immunoreactivity for p63 [29]. While normal skin showed strong nuclear p63 immunoreactivity as an internal positive control, only a small number of scattered cells in tumours in 129/Sv mice displayed weak cytoplasmic staining for p63 (Figure 4G), arguing against a diagnosis of sarcomatoid squamous cell carcinoma. Tumours were also completely negative for the epithelial marker EpCAM (Figure 4H), but scattered cells within the 129/Sv subcutaneous tumours (Figure 4I), as well as rare cells in angiosarcomas in SCID/beige mice (Figure 4J), reacted with a pan-CYTOKERATIN antibody, another epithelial marker. However, since human sarcomas, including UPS and angiosarcoma, can contain cells that are immunoreactive for pan-CYTOKERATIN caution should be taken in interpreting this staining as providing strong evidence of epithelial origin [30]. Positive control stainings of all of these antibodies are shown in Supplementary Figure 4.

Given the absence of morphologic features of any cellular lineage, the absence of clear evidence for positivity of tumour cells for a panel of lineage markers and absence of strong and diffuse pan-CYTOKERATIN staining we therefore favour the diagnosis of these tumours as high-grade UPS Not Otherwise Specified in keeping with WHO diagnostic guidelines. However, since the differential diagnosis of UPS versus sarcomatoid carcinoma is necessarily a diagnosis of exclusion, it remains formally possible that these tumours might represent an unknown type of undifferentiated sarcomatoid carcinoma arising from a cell type that we have not been able to identify. As already known from human tumour specimens, the differential diagnosis of these lesions remains a continuous matter of debate. Importantly, these analyses show that 129/Sv mice develop two different types of tumours, angiosarcomas and UPS, in response to the same oncogenic stimulus.

**Figure 5:**
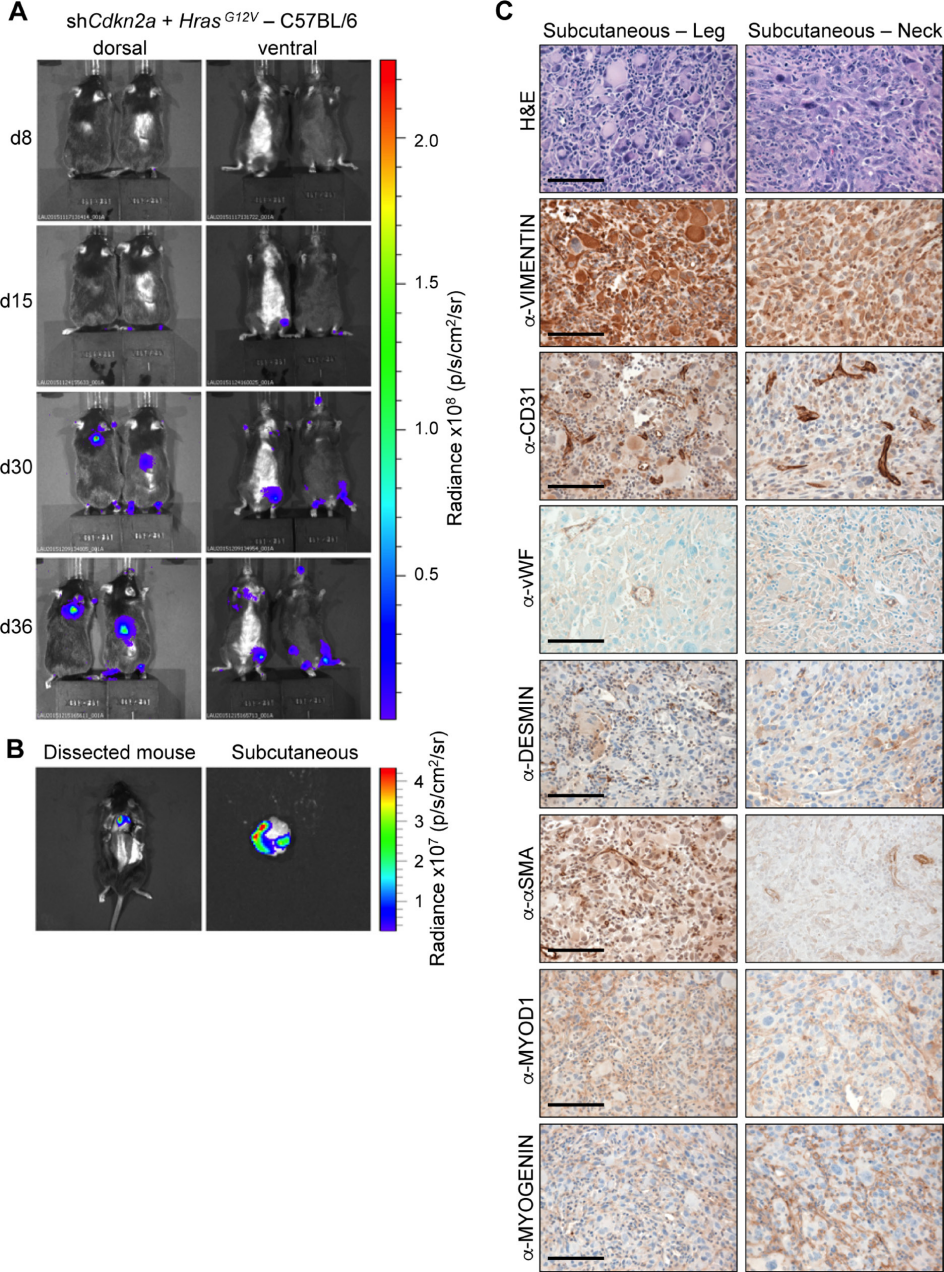
*Hras^G12V^* expression plus knockdown of *Cdkn2a* causes undifferentiated pleomorphic sarcomas in C57BL/6 mice (**A**) Bioluminescence imaging 8, 15, 30 and 36 days after the injection of MuLE lentiviruses expressing shRNA against *Cdkn2a* together with *Hras^G12V^* into the tail veins of 4-6-week-old C57Bl/6 mice. (**B**) Bioluminescence imaging showing examples of a subcutaneous tumour prior to (left) and after (right) resection. (**C**) H&E and immunohistochemical stainings using the indicated antibodies. Scale bars: 100 μm.

To further investigate the effect of genetic background on tumour formation, we injected C57BL/6 mice with sh*Cdkn2a* plus *Hras^G12V^* MuLE lentiviruses. Within 4–8 weeks of injection both male and female C57BL/6 mice showed large increases in luciferase signal and developed subcutaneous lesions with 92% penetrance (12 of 13 injected mice) (Figure 5A and 5B). Tumours (*n* = 12) that developed in C57BL/6 mice were solid and white in appearance like those seen in female 129/Sv mice. They were subcutaneous and located either in the head and neck area, lower leg or at the junction of the tail and spine. These tumours exhbited an identical histological appearance to the UPS tumours that arose in 129/Sv mice (Figure 4B). These tumours similarly showed an absence of staining for all of the markers that were used to characterise the tumours in 129Sv mice, except VIMENTIN (Figure 5C). Based on these results we conclude that sh*Cdkn2a* plus *Hras^G12V^* MuLE viruses solely cause high-grade UPS in C57BL/6 mice.

## DISCUSSION

Major hurdles in studying sarcoma pathologies are the relative rarities of the human diseases and the absence for many sarcoma subtypes of good pre-clinical models. Here, we used the MuLE lentiviral gene regulatory system [10] to investigate the molecular genetics underlying the pathogenesis of two types of soft tissue sarcomas, namely angiosarcoma and UPS. The MuLE system allows the direct introduction of multiple genetic alterations in somatic cells *in vivo* by lentiviral injection. Bypassing germline transgenic approaches has benefits in terms of time and costs and offers flexibility in terms of the strains of mice that can be used for the experiments, allowing comparisons between different genetic backgrounds. Guided by the genetics of human angiosarcomas and UPS tumours, we functionally tested the contributions of the candidate sarcoma tumour suppressors *Cdkn2a*, *Trp53, Tsc2* and *Pten* and the candidate oncogenes *Hras^G12V^*, *PIK3CA^H1047R^* or *Myc*. We discovered that the systemic injection of ecotropic lentiviruses expressing oncogenic *Hras^G12V^* together with the knockdown of *Cdkn2a* or *Trp53* was sufficient to initiate angiosarcoma formation in multiple organs in SCID/beige mice. sh*Cdkn2a* plus *Hras^G12V^* MuLE viruses also induced angiosarcoma formation in Fox Chase CB17 and 129/Sv mice, but surprisingly additionally caused UPS development in 129/Sv mice and only UPS development in C57/BL6 mice. These observations are consistent with the fact that RAS-MAPK and p53 pathway alterations are frequently found in high-grade soft tissue sarcomas, such as angiosarcomas and UPS [2, 3, 6, 7, 19].

Our experimental approach provides new models of at least some of the genetic subsets of human angiosarcomas and UPS tumours. It should also be noted that while histological and immunohistochemical analyses revealed numerous similarities between mouse and human tumours, there are differences in terms of the anatomical sites where the mouse model tumours arise and the most frequent sites where naturally occurring human counterpart tumours arise. Mouse angiosarcomas in our model arose most frequently in the brain, genital organs and spleen. Human angiosarcomas are most frequently found in the skin (60%), deep soft tissue (25%), breast (8%) and more rarely in liver and spleen [1]. There are also examples of cases of human angiosarcomas arising in the testes [31], uterus and ovaries [32], brain [33], lungs [34] and colon [35], which are all sites at which tumours were observed in our mouse models. Since endothelial cells are present in all tissues in the body, it appears that angiosarcomas have the potential to arise in any organ in humans. In our mouse model, the anatomical distribution of tumours is likely to be influenced by the method of intravascular injection, which will presumably influence the likelihood of viral infection at different sites in the animal. In contrast, UPS tumours in our models were always localised subcutaneously. In humans UPS can arise anywhere in the body but most frequently occurs in lower extremeties and sometimes in the retroperitoneum, head and neck and breast. These tumours typically arise in deep subfascial tissue, but about 10% of UPS are primarily subcutaneous [1], similarly to the tumours that arise in our mouse models.

Genetic cooperation between RAS pathway activation and loss of *Cdkn2a* tumour suppressor function has been shown in other mouse models of sarcomas. Intramuscular injection of Adeno-Cre in the leg of *loxP-STOP-loxP-Kras*^G12D/+^;*Cdkn2a*^fl/fl^ mice caused UPS development [9, 11] and we have previously shown that intramuscular injection of the same sh*Cdkn2a* plus *Hras^G12V^* or sh*Trp53* plus *Hras^G12V^* MuLE lentiviruses that were used in this study caused the developement of high-grade UPS [10]. It is noteworthy that injection of these viruses into skeletal muscle in SCID-beige mice caused UPS [10] but intravenous injection caused angiosarcoma, whereas in C57/BL6 mice both modes of injection caused UPS formation [10]. Our data demonstrate that the same combination of genetic drivers can cause different types of tumours based not only on the site of viral delivery, likely due to infection of different cell types, but also based on the genetic background of the mouse strain.

Why does intraveneous, lentiviral-mediated delivery of the same genetic alterations cause different tumours in different strains of mice? One possibility relates to genetic modifiers such as allelic variants, sequence differences, epigenetic modifications and gene expression levels that could potentially influence tumour phenotypes [36]. There is precedent for the fact that identical genetic alterations can result in different phenotypes in different mouse strains. One example is that the incidence of mammary tumours varies among strains heterozygous for *Trp53*, with C57BL/6 mice being resistant and BALB/c mice being susceptible [37, 38]. Indeed, BALB/c mice carry an allelic variant of *Cdkn2a* that leads to compromised p16 activity, likely causing an increased susceptibility to develop certain tumours [39, 40]. In the context of the results of the present study, it might be possible in future long-term and large-scale studies to utilise multi-generational interbreeding strategies between C57BL/6 and Fox Chase CB17 strains, coupled with genomic analyses, to narrow down loci that contribute to the type of sarcoma that develops in response to oncogenic *Hras^G12V^* expression and *Cdkn2a* knockdown. By extension, our observations highlight the fact that also in humans the genetic background of every individual may potentially influence the outcome of oncogenic mutations in terms of what type of sarcoma develops. In this context, it is noteworthy that a study of UPS tumours in Korean patients identified a high frequency of oncogenic *KRAS* and *HRAS* mutations [6], but a study by the same authors of UPS tumours in American patients revealed that these tumours lacked *KRAS* and *HRAS* mutations [7], arguing that different human genetic backgrounds (or environmental factors) may select for different oncogenic mutations during the course of sarcoma development. To further investigate this issue, we analysed the mutation spectrum of a series of 48 human UPS tumours from the TCGA provisional dataset using cBioPortal software (http://www.cbioportal.org/study?id=sarc_tcga#summary) (Supplementary Figure 7). The ethnicity of this patient cohort is 42 white, 4 black or African American, 2 hispanic or latino, 2 unknown but no reported Asian patients. Interestingly, this set of UPS tumours does not display activating point mutations in any of the RAS-family genes in keeping with the previous study of American patients [7]. However, the majority of these tumours exhibit loss-of-function point mutations or copy number deletions of the *TP53* and *CDKN2A* tumour suppressor genes and all but one of these tumours exhibit multiple copy number gains or amplifications of genes involved in the RAS-RAF-MEK-MAPK signalling cascade, as well as copy number losses of the *NF1* tumour suppressor gene that negatively regulates signalling by this cascade. These results give rise to a hypothesis that could be tested in future studies, namely that chromosomal copy number alterations rather than point mutations account for activation of the RAS-signalling pathway in combination with loss of the CDKN2A-TP53 tumour suppressor pathways to drive UPS tumour formation in non-Asian individuals. In summary, we believe that our functional tumour modelling studies, combined with the above-described genetic analyses, further emphasise the need to consider individual tumours at the molecular level rather than at the level of histo-pathological appearance when thinking about the development and clinical application of new molecularly targeted sarcoma therapies.

A second possible explanation for the different tumour types that arise in the different mouse stains relates to cellular tropism. It remains possible that MuLE viruses might infect different spectra of cells in different mouse strains, potentially due to different expression levels of the mouse cationic amino acid transporter 1 (mCAT1) protein that serves as the virus receptor. However, to our knowledge, nothing is known about the expression patterns of this protein in different strains of mice and in our experience identifying infected cells *in vivo* is only possible using genetic reporter mouse lines [10]. However, the identification of infection of a particular cell type is at best a hint that it might be able to be transformed and act as the cell of origin of a tumour. Consistent with the idea that angiosarcoma and UPS tumours might arise due to the infection of different cell types it is noteworthy that all of the UPS tumours that arose in 129/Sv and C57BL/6 mice were subcutaneous, whereas angiosargomas in 129/Sv, Fox Chase CB17 and SCID/beige mice were found in several different organs. These differences in anatomical distribution are suggestive of different cells of origin of these tumours. Proof of cell of orgin of a tumour requires a lineage tracing experiment. Given the fact that angiosarcomas express markers of endothelial cells and are intimately connected to the normal vascular network of the mouse (blood-filled lesions) it appears likely that the cell of origin of these tumours are vascular endothelial cells. Future experiments could involve utilising lineage tracing experiments using a Cre-driver such as Tie2-Cre^ERT2^ [41] to genetically label endothelial cells prior to injection of sh*Cdkn2a* plus *Hras^G12V^* MuLE lentiviruses. A potential caveat of these studies may however be that the tumour type that will arise will also be dictated by the (likely mixed) genetic background of the strain of the lineage tracing mouse. The cell of origin of UPS remains unknown and it is not clear whether UPS represent a group of de-differentiated sarcomas which share a common morphology but originated from different cell types or if all UPS tumours arise from a common cell of origin [5]. One interesting hypothesis that could be experimentally tested is that adult mesenchymal stem cells (MSCs) have been proposed as the cell of origin of human UPS [42–45]. It has been shown that the overexpression of oncogenic *Kras* in cultured MSCs isolated from the bone marrow of *Trp53* knockout C57BL/6 mice caused transformation of these cells *in vitro* [46]. We have shown that ecotropic MuLE lentiviruses can infect a variety of cultured primary cells, including mouse embryonic stem cells [10] and although bone marrow represents the main source of MSCs, MSCs also exist in low numbers in peripheral blood [47]. It is theoretically possible that infected MSCs in the peripheral blood could give rise to UPS in 129/Sv and C57BL/6 mice.

In summary, these new experimental models will facilitate future pre-clinical studies for establishing new therapeutic interventions for these aggressive malignancies. While it is somewhat surprising that the other tested candidate tumour suppressors and oncogenes were not sufficient to cause tumour formation, given their frequent mutational alteration in human angiosarcomas and UPS, the flexible nature of the MuLE system should also allow the testing of other candidate oncogenes, modifier genes or tumour suppressor genes that will likely continue to emerge from ongoing genomic studies of these rare tumours.

## MATERIALS AND METHODS

### Mice

SCID/beige mutant mice (C.B-17/CrHsd-Prkdc^Scid^Lyst^bg-J^) and C57BL/6JRccHsd mice were obtained from Envigo. 129S2/SvPasOrlRj mice were obtained from Janvier Labs and Fox Chase CB17™ mice (C.BKa-lgh^b^/lcrCrl) were obtained from Charles River Laboratories. All mouse experiments were approved by the Veterinary Office of the Canton of Zurich under the licence 137/2013.

### Generation of MuLE vectors

The majority of the MuLE Entry and Destination vectors used in this study were previously described [10] and were recombined using MultiSite Gateway LR 2-fragment recombinations to generate the final viral constructs. New MuLE Entry vectors carrying 7SK promotor-driven expression of shRNA against *Tsc2* (Sigma, TRCN0000306244) and CMV promotor-driven expression of hemagglutinin (HA) tagged phosphoinositide 3-kinase H1047R (HA-PIK3CA H1047R, Addgene 12524, deposited by Dr. Jean Zhao) were generated. Ecotropic lentiviral vectors were produced by using calcium phosphate-mediated transfection of HEK293T cells and the viral preparation was concentrated as described previously [10].

### Culture, infection and assays of pMSECs

C57BL/6 mouse primary spleen endothelial cells (pMSECs, C57-6057, Cell Biologics) were cultured according to the instructions of the supplier in complete mouse endothelial cell medium (M1168, Cell Biologics) in a humidified 5% (v/v) CO_2_ incubator at 37°C. 50% confluent pMSECs were transduced with lentiviral vectors in the presence of 4 μg/ml polybrene (Hexadimethrine bromide, 107689, Sigma-Aldrich). Puromycin (ANT-PR-1, InvivoGen) was added to cultures 48 h after infection and drug selection continued until all control cells were dead. pMSECs were seeded in triplicates at density of 2,000 cells per well in 96-well plates and analysed after 1, 3, 5 and 7 days using the SRB assay [48]. For allograft assays, 1 × 10^6^ pMSECs were injected subcutaneously into SCID/beige mice.

### *In vivo* tumour formation assays

Concentrated ecotropic lentiviruses were injected (3 × 10^5^ infectious viral particles in a volume of 10 ml/kg) using a 30G insulin syringe into the lateral tail vein of 4–6-week old mice. Noninvasive *in vivo* bioluminescence imaging was performed using the IVIS Spectrum (Perkin Elmer) together with the Living Image software (version 4.4). Mice were anaesthetized by using 2.5% isoflurane. During imaging, the isoflurane levels were reduced to 1.5%. All fluorescence measurements were performed in epi-fluorescence mode. For bioluminescence imaging, mice were injected subcutaneously with 150 mg/kg D-luciferin (Caliper, no. 122796) and imaged 15 min after injection.

### Immunohistochemistry

Tumour-bearing organs were resected, fixed in 10% formalin, paraffin-embedded and cut in 5 μm thick sections. Immunohistochemical analysis was performed as described [49]. The antibodies used in this study were anti-CD31 (1:200, ab28364, Abcam), anti-DESMIN (1:100, D1033, Sigma-Aldrich), anti-EpCAM (1:500, ab71916, Abcam), anti-H-RAS (1:100, GTX116041, GeneTex), anti-MYOD1 (1:100, M3512, Dako), anti-MYOGENIN (1:500, M3559, Dako), anti-pan-CYTOKERATIN (1:100, BMA Biomedicals AG, T1302), anti-PMEL(HMB45) (1:100, Abcam, ab137078), anti-p63 (1:500, ab124762, Abcam), anti-alpha-SMA (1:5000, ab5694, Abcam), anti-S100 (1:2000, S100 DAKO A/S), anti-VIMENTIN (1:500, D21H3, Cell Signaling) and anti-von Willebrand Factor (vWF) (1:1000, F3520, Sigma-Aldrich).

### Western blotting

Cells were lysed in RIPA buffer (50 mM Tris-HCl pH 8.0, 150 mM sodium chloride, 1% (v/v) NP-40, 0.5% (w/v) sodium deoxycholate, 0.1% (w/v) SDS, 5mM sodium fluoride, 1 mM sodium orthovanadate, 1 mM PMSF, 1:100 Protease Inhibitor Cocktail (Sigma-Aldrich)) for protein extraction. Proteins were run on 8–12% acrylamide gels, transferred to nitrocellulose membranes and visualized by immunoblotting with the following antibodies anti-ß-ACTIN (1:2000, A2228, Sigma-Aldrich), anti-H-RAS (1:1000, sc-520, Santa Cruz Biotechnology Inc.), anti-MYC (1:1000, AB32072, Epitomics (Abcam)), anti-p16 (1:1000, sc-1207, Santa Cruz Biotechnology Inc.), anti-p19 (1:1000, sc-32748, Santa Cruz Biotechnology Inc.), anti-p53 (1:500, NCL-p53-CM5p, Novocastra), anti-PTEN (1:1000, sc-7974, Santa Cruz Biotechnology Inc.), anti-TSC2 (1:1000, 3990s, Cell signaling) and anti-VINCULIN (1:5000, ab129002, Abcam).

### Real-time PCR

Total RNA was isolated from cells using NucleoSpin RNA kit (Macherey-Nagel). cDNA systhesis was performed with random hexamer primers and Ready-To-Go You-Prime First-Strand Beads (GE Healthcare). Real-time PCR analysis was performed with SYBR Green JumpStart Taq Ready Mix (Sigma-Aldrich) and the following primer pairs: *S12* (5′-GAAGCTGC CAAAGCCTTAGA-3′, 5′-AACTGCAACCAACCACC TTC-3′) and *PIK3CA* (5′- CCACGACCATCATCAGGT GAA-3′, 5′- CCTCACGGAGGCATTCTAAAGT-3′).

## SUPPLEMENTARY MATERIALS FIGURES



## References

[1] Fletcher C (2013). WHO classification of tumours of soft tissue and bone. Fourth Edition.

[2] Barretina J, Taylor BS, Banerji S, Ramos AH, Lagos-Quintana M, Decarolis PL, Shah K, Socci ND, Weir BA, Ho A, Chiang DY, Reva B, Mermel CH (2010). Subtype-specific genomic alterations define new targets for soft-tissue sarcoma therapy. Nat Genet.

[3] Pérot G, Chibon F, Montero A, Lagarde P, de Thé H, Terrier P, Guillou L, Ranchère D, Coindre JM, Aurias A (2010). Constant p53 Pathway Inactivation in a Large Series of Soft Tissue Sarcomas with Complex Genetics. The American Journal of Pathology.

[4] Borden EC, Baker LH, Bell RS, Bramwell V, Demetri GD, Eisenberg BL, Fletcher CD, Fletcher JA, Ladanyi M, Meltzer P, O'Sullivan B, Parkinson DR, Pisters PW (2003). Soft tissue sarcomas of adults: state of the translational science. Clin Cancer Res.

[5] Matushansky I, Charytonowicz E, Mills J, Siddiqi S, Hricik T, Cordon-Cardo C (2009). MFH classification: differentiating undifferentiated pleomorphic sarcoma in the 21st Century. Expert Rev Anticancer Ther.

[6] Yoo J, Robinson RA, Lee JY (1999). H-ras and K-ras gene mutations in primary human soft tissue sarcoma: concomitant mutations of the ras genes. Mod Pathol.

[7] Yoo J, Robinson RA (1999). H-ras and K-ras mutations in soft tissue sarcoma. Cancer.

[8] Bohle RM, Brettreich S, Repp R, Borkhardt A, Kosmehl H, Altmannsberger HM (1996). Single somatic ras gene point mutation in soft tissue malignant fibrous histiocytomas. The American Journal of Pathology.

[9] Kirsch DG, Dinulescu DM, Miller JB, Grimm J, Santiago PM, Young NP, Nielsen GP, Quade BJ, Chaber CJ, Schultz CP, Takeuchi O, Bronson RT, Crowley D (2007). A spatially and temporally restricted mouse model of soft tissue sarcoma. Nature Medicine.

[10] Albers J, Danzer C, Rechsteiner M, Lehmann H, Brandt LP, Hejhal T, Catalano A, Busenhart P, Gonçalves AF, Brandt S, Bode PK, Bode-Lesniewska B, Wild PJ (2015). A versatile modular vector system for rapid combinatorial mammalian genetics. J Clin Invest.

[11] Hettmer S, Liu J, Miller CM, Lindsay MC, Sparks CA, Guertin DA, Bronson RT, Langenau DM, Wagers AJ (2011). Sarcomas induced in discrete subsets of prospectively isolated skeletal muscle cells. Proc Natl Acad Sci U S A.

[12] Blum JM, Añó L, Li Z, Van Mater D, Bennett BD, Sachdeva M, Lagutina I, Zhang M, Mito JK, Dodd LG, Cardona DM, Dodd RD, Williams N (2013). Distinct and Overlapping Sarcoma Subtypes Initiated from Muscle Stem and Progenitor Cells. Cell Reports.

[13] Young RJ, Brown NJ, Reed MW, Hughes D, Woll PJ (2010). Angiosarcoma. Lancet Oncol.

[14] Zietz C, Rössle M, Haas C, Sendelhofert A, Hirschmann A, Stürzl M, Löhrs U (1998). MDM-2 Oncoprotein Overexpression, p53 Gene Mutation, and VEGF Up-Regulation in Angiosarcomas. The American Journal of Pathology.

[15] Manner J, Radlwimmer B, Hohenberger P, Mössinger K, Küffer S, Sauer C, Belharazem D, Zettl A, Coindre JM, Hallermann C, Hartmann JT, Katenkamp D, Katenkamp K (2010). MYC High Level Gene Amplification Is a Distinctive Feature of Angiosarcomas after Irradiation or Chronic Lymphedema. The American Journal of Pathology.

[16] Weihrauch M, Bader M, Lehnert G, Koch B, Wittekind C, Wrbitzky R, Tannapfel A (2002). Mutation analysis of K-ras-2 in liver angiosarcoma and adjacent nonneoplastic liver tissue from patients occupationally exposed to vinyl chloride. Environmental and Molecular Mutagenesis.

[17] Hayashi T, Koike K, Kumasaka T, Saito T, Mitani K, Terao Y, Ogishima D, Yao T, Takeda S, Takahashi K, Seyama K (2012). Uterine angiosarcoma associated with lymphangioleiomyomatosis in a patient with tuberous sclerosis complex: an autopsy case report with immunohistochemical and genetic analysis. Human Pathology.

[18] Guo T, Zhang L, Chang NE, Singer S, Maki RG, Antonescu CR (2011). Consistent MYC and FLT4 gene amplification in radiation-induced angiosarcoma but not in other radiation-associated atypical vascular lesions. Genes Chromosomes Cancer.

[19] Murali R, Chandramohan R, Möller I, Scholz SL, Berger M, Huberman K, Viale A, Pirun M, Socci ND, Bouvier N, Bauer S, Artl M, Schilling B (2015). Targeted massively parallel sequencing of angiosarcomas reveals frequent activation of the mitogen activated protein kinase pathway. Oncotarget.

[20] Behjati S, Tarpey PS, Sheldon H, Martincorena I, Van Loo P, Gundem G, Wedge DC, Ramakrishna M, Cooke SL, Pillay N, Vollan HKM, Papaemmanuil E, Koss H (2014). Recurrent PTPRB and PLCG1 mutations in angiosarcoma. Nat Genet.

[21] Landuzzi L, Ianzano ML, Nicoletti G, Palladini A, Grosso V, Ranieri D, Dall’Ora M, Raschi E, Laranga R, Gambarotti M, Picci P, De Giovanni C, Nanni P (2014). Genetic prevention of lymphoma in p53 knockout mice allows the early development of p53-related sarcomas. Oncotarget.

[22] Ghahremani MF, Radaelli E, Haigh K, Bartunkova S, Haenebalcke L, Marine JC, Goossens S, Haigh JJ (2014). Loss of autocrine endothelial-derived VEGF significantly reduces hemangiosarcoma development in conditional p53-deficient mice. Cell Cycle.

[23] Papa A, Cordon-Cardo C, Bernardi R, Pandolfi PP (2012). Compound In Vivo Inactivation of Pml and p53 Uncovers a Functional Interaction in Angiosarcoma Suppression. Genes Cancer.

[24] Yang J, Kantrow S, Sai J, Hawkins OE, Boothby M, Ayers GD, Young ED, Demicco EG, Lazar AJ, Lev D, Richmond A (2012). Ikk4a/Arf Inactivation with Activation of the NF-κB/IL-6 Pathway Is Sufficient to Drive the Development and Growth of Angiosarcoma. Cancer Res.

[25] Italiano A, Chen CL, Thomas R, Breen M, Bonnet F, Sevenet N, Longy M, Maki RG, Coindre JM, Antonescu CR (2012). Alterations of the p53 and PIK3CA/AKT/mTOR pathways in angiosarcomas: a pattern distinct from other sarcomas with complex genomics. Cancer.

[26] Tate G, Suzuki T, Mitsuya T (2007). Mutation of the PTEN gene in a human hepatic angiosarcoma. Cancer Genetics and Cytogenetics.

[27] Leech JD, Lammers SH, Goldman S, Auricchio N, Bronson RT, Kwiatkowski DJ, Sahin M (2015). A Vascular Model of Tsc1 Deficiency Accelerates Renal Tumor Formation with Accompanying Hemangiosarcomas. Mol Cancer Res.

[28] Dill MT, Rothweiler S, Djonov V, Hlushchuk R, Tornillo L, Terracciano L, Meili Butz S, Radtke F, Heim MH, Semela D (2012). Disruption of Notch1 Induces Vascular Remodeling, Intussusceptive Angiogenesis, and Angiosarcomas in Livers of Mice. Gastroenterology.

[29] Hall JM, Saenger JS, Fadare O (2008). Diagnostic utility of P63 and CD10 in distinguishing cutaneous spindle cell/sarcomatoid squamous cell carcinomas and atypical fibroxanthomas. Int J Clin Exp Pathol.

[30] Goldblum JR (2014). An approach to pleomorphic sarcomas: can we subclassify, and does it matter?. Mod Pathol.

[31] Armah HB, Rao UN, Parwani AV (2007). Primary angiosarcoma of the testis: report of a rare entity and review of the literature. Diagn Pathol.

[32] Kruse AJ, Sep S, Slangen BF, Vandevijver NM, Van Gorp T, Kruitwagen RF, Van de Vijver KK (2014). Angiosarcomas of primary gynecologic origin: a clinicopathologic review and quantitative analysis of survival. Int J Gynecol Cancer.

[33] Hackney JR, Palmer CA, Riley KO, Cure JK, Fathallah-Shaykh HM, Nabors LB (2012). Primary central nervous system angiosarcoma: two case reports. J Med Case Rep.

[34] Ren Y, Zhu M, Liu Y, Diao X, Zhang Y (2016). Primary pulmonary angiosarcoma: Three case reports and literature review. Thorac Cancer.

[35] Al Beteddini OS, Brenez D, Firket C, Algaba R, Tabech A (2013). Colonic angiosarcoma: A case report and review of literature. Int J Surg Case Rep.

[36] Sigmund CD (2000). Viewpoint: are studies in genetically altered mice out of control?. Arterioscler Thromb Vasc Biol.

[37] Medina D (1974). Mammary tumorigenesis in chemical carcinogen-treated mice. I. Incidence in BALB-c and C57BL mice. J Natl Cancer Inst.

[38] Ullrich RL, Bowles ND, Satterfield LC, Davis CM (1996). Strain-dependent susceptibility to radiation-induced mammary cancer is a result of differences in epithelial cell sensitivity to transformation. Radiat Res.

[39] Zhang S, Qian X, Redman C, Bliskovski V, Ramsay ES, Lowy DR, Mock BA (2003). p16INK4a gene promoter variation and differential binding of a repressor, the ras-responsive zinc-finger transcription factor, RREB. Oncogene.

[40] Blackburn AC, Brown JS, Naber SP, Otis CN, Wood JT, Jerry DJ (2003). BALB/c alleles for Prkdc and Cdkn2a interact to modify tumor susceptibility in Trp53+/– mice. Cancer Res.

[41] Forde A, Constien R, Gröne HJ, Hämmerling G, Arnold B (2002). Temporal Cre-mediated recombination exclusively in endothelial cells using Tie2 regulatory elements. Genesis.

[42] Rodriguez R, Rubio R, Menendez P (2011). Modeling sarcomagenesis using multipotent mesenchymal stem cells. Cell Research.

[43] Mohseny AB, Hogendoorn PCW (2011). Concise review: mesenchymal tumors: when stem cells go mad. Stem Cells.

[44] Rubio R, García-Castro J, Gutiérrez-Aranda I, Paramio J, Santos M, Catalina P, Leone PE, Menendez P, Rodriguez R (2010). Deficiency in p53 but not Retinoblastoma Induces the Transformation of Mesenchymal Stem Cells In vitro and Initiates Leiomyosarcoma In vivo. Cancer Res.

[45] Tolar J, Nauta AJ, Osborn MJ, Panoskaltsis Mortari A, McElmurry RT, Bell S, Xia L, Zhou N, Riddle M, Schroeder TM, Westendorf JJ, McIvor RS, Hogendoorn PCW (2007). Sarcoma derived from cultured mesenchymal stem cells. Stem Cells.

[46] Guarnerio J, Riccardi L, Taulli R, Maeda T, Wang G, Hobbs RM, Song MS, Sportoletti P, Bernardi R, Bronson RT, Castillo-Martin M, Cordon-Cardo C, Lunardi A (2015). A genetic platform to model sarcomagenesis from primary adult mesenchymal stem cells. Cancer Discov.

[47] He Q, Wan C, Li G (2007). Concise review: multipotent mesenchymal stromal cells in blood. Stem Cells.

[48] Vichai V, Kirtikara K (2006). Sulforhodamine B colorimetric assay for cytotoxicity screening. Nature Protocols.

[49] Thoma CR, Frew IJ, Hoerner CR, Montani M, Moch H, Krek W (2007). pVHL and GSK3β are components of a primary cilium-maintenance signalling network. Nat Cell Biol.

