# Supplementary material for: Mouse genetic background influences whether HrasG12V expression plus Cdkn2a knockdown causes angiosarcoma or undifferentiated pleomorphic sarcoma
Source: Oncotarget. 2018 Apr 13;9(28):19753–66. doi: 10.18632/oncotarget.24831 (PMC5929423; doi:10.18632/oncotarget.24831)
Supplement: Supplementary file 1 [file oncotarget-09-19753-s001.pdf]

Mouse genetic background influences whether *Hras^{G12V}* expression plus *Cdkn2a* knockdown causes angiosarcoma or undifferentiated pleomorphic sarcoma

SUPPLEMENTARY MATERIALS

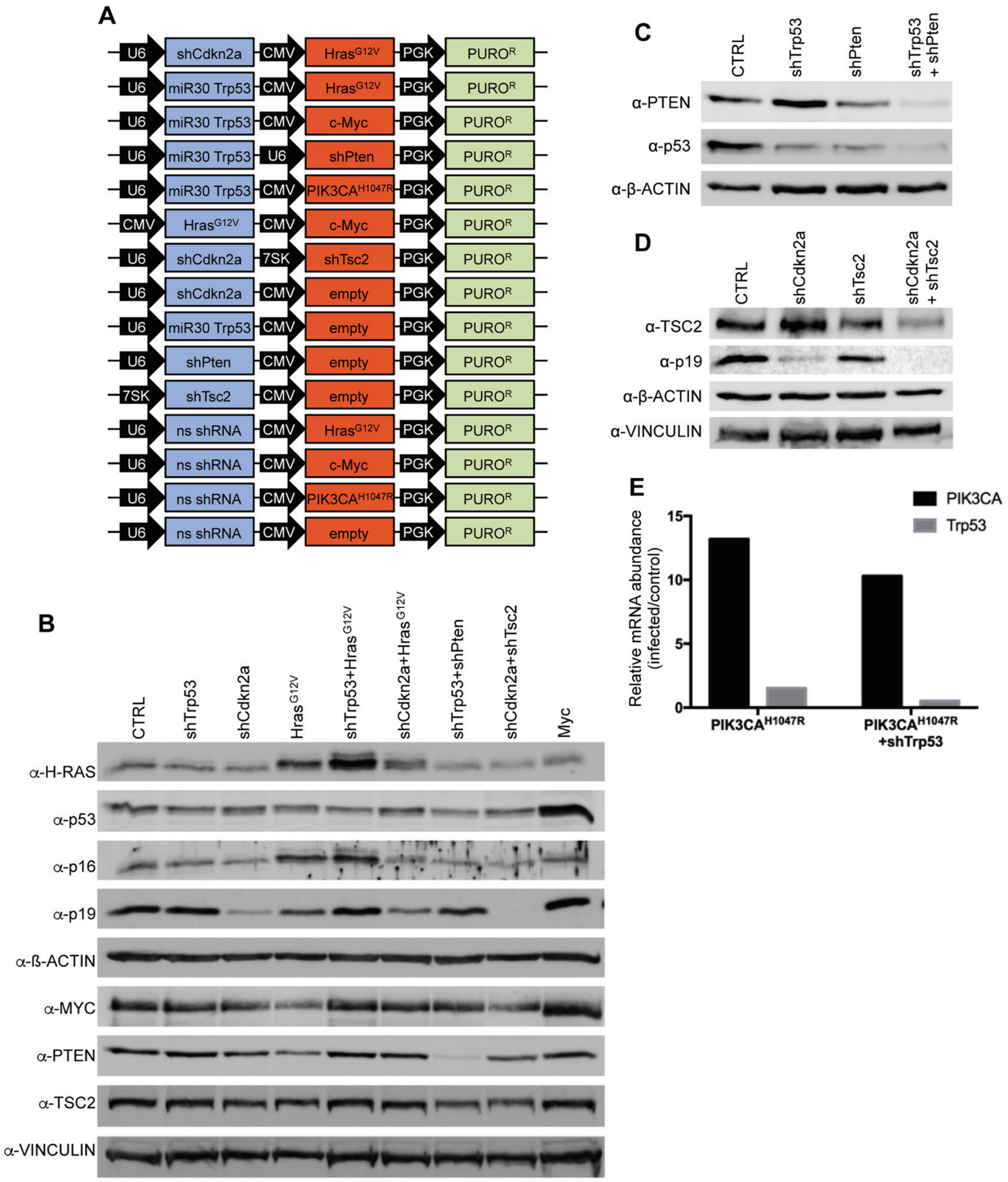

Supplementary Figure 1: Validation of MuLE vectors by western blot and real time PCR analysis. (**A**) Schematic of MuLE vectors simultaneously expressing combinations of shRNAs against *Cdkn2a*, *Trp53*, *Pten* and *Tsc2* with or without expression of *Hras^{G12V}*, *Myc* or *PIK3CA^{H1047R}* and puromycin resistance. (**B–D**) Western blots of primary endothelial cells from the spleen (pMSECs) transduced with the indicated lentiviruses. Control (CTRL) refers to the final vector depicted in A expressing a non-silencing control shRNA and lacking a cDNA behind the CMV promoter. (**E**) Relative gene expression of *PIK3CA* and *Trp53* in pMSECs transduced with the indicated lentiviruses.

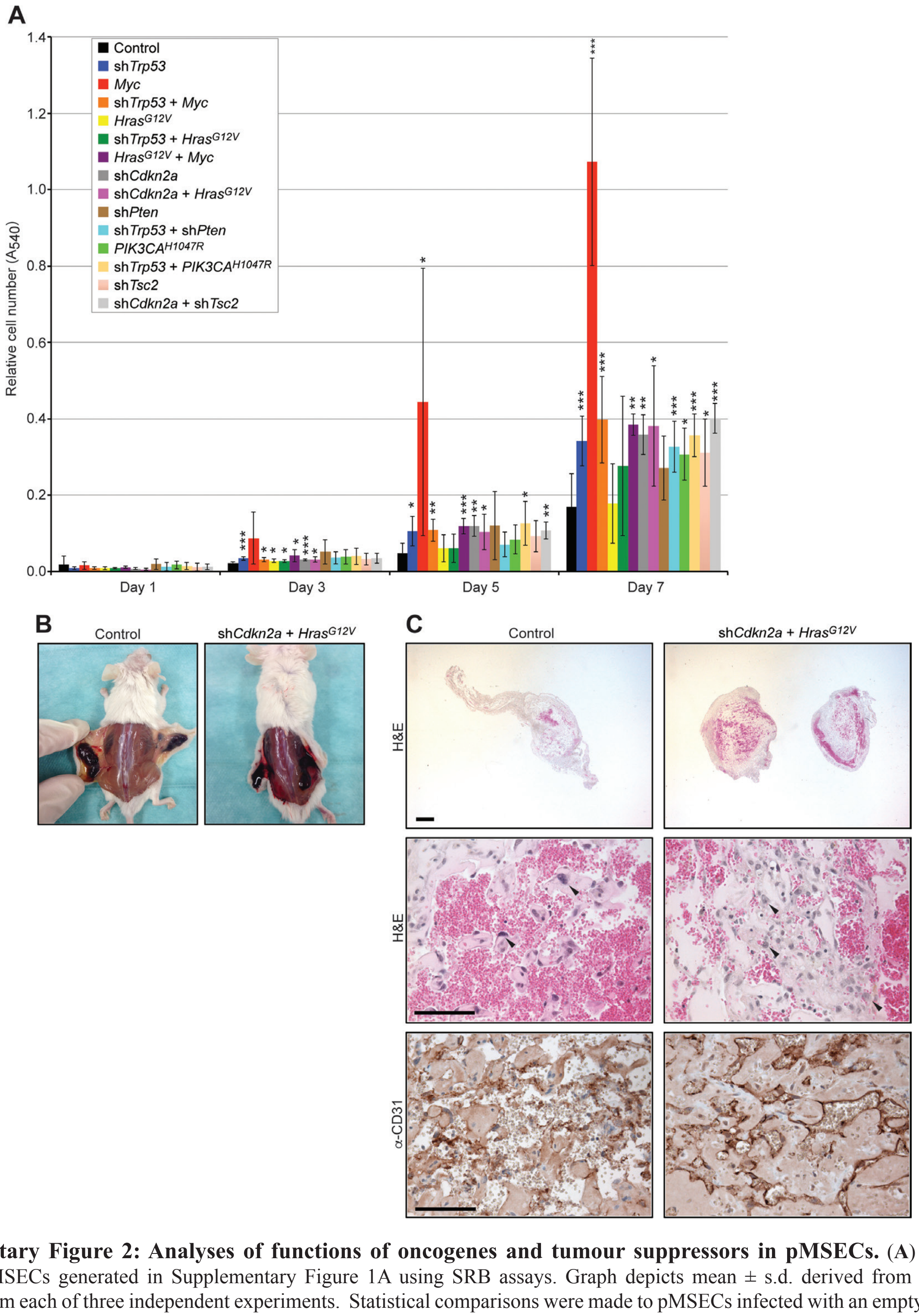

Supplementary Figure 2: Analyses of functions of oncogenes and tumour suppressors in pMSECs. (**A**) Proliferation assays of pMSECs generated in Supplementary Figure 1A using SRB assays. Graph depicts mean ± s.d. derived from experimental triplicates from each of three independent experiments. Statistical comparisons were made to pMSECs infected with an empty MuLE virus (Control) using Student's *t* test. $^{*}P < 0.05$, $^{**}P < 0.01$, $^{***}P < 0.01$. (**B**) Images of blood-filled lesions formed 4 weeks after subcutaneous injection of control pMSECs or pMSECs infected with MuLE lentiviruses expressing a shRNA against *Cdkn2a* together with $Hras^{G12V}$. (**C**) H&E and anti-CD31 immunohistochemical staining of the lesions described in B. Arrowheads highlight aberrant nuclei.

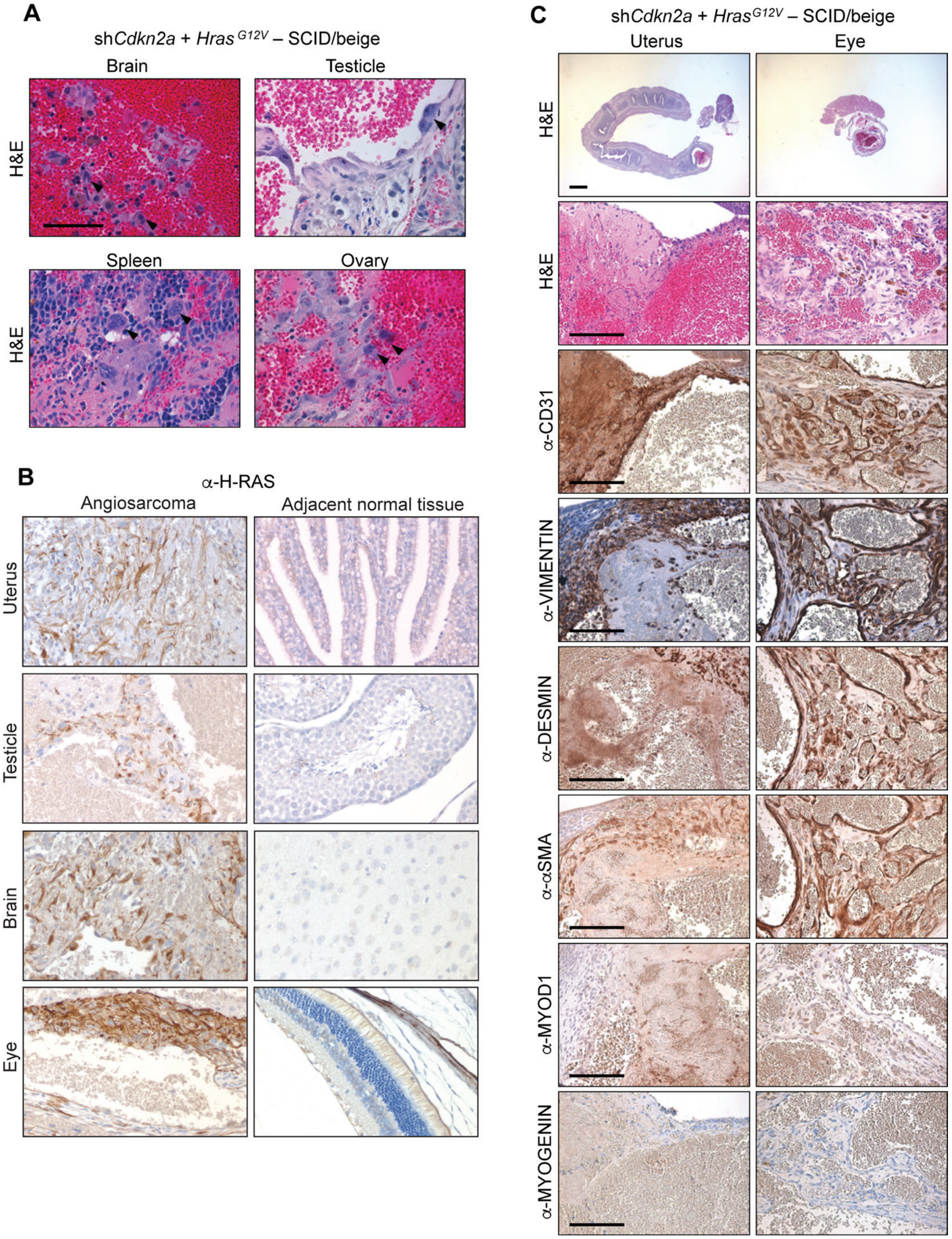

Supplementary Figure 3: Additional information related to Figure 1 and Figure 2. (**A**) High magnification images of H&E stained tumour sections derived from the systemic injection of sh*Cdkn2a* plus $Hras^{G12V}$ viruses in SCID/beige mice. Arrowheads indicate atypical nuclei. Scale bar: 100 µm. (**B**) Immunohistochemical stainings using an anti-H-RAS antibody of sh*Cdkn2a* plus $Hras^{G12V}$ angiosarcomas and adjacent normal tissue. (**C**) H&E and immunohistochemical stainings of additional tumours derived from the systemic injection of sh*Cdkn2a* plus $Hras^{G12V}$ viruses. Scale bars: 100 µm.

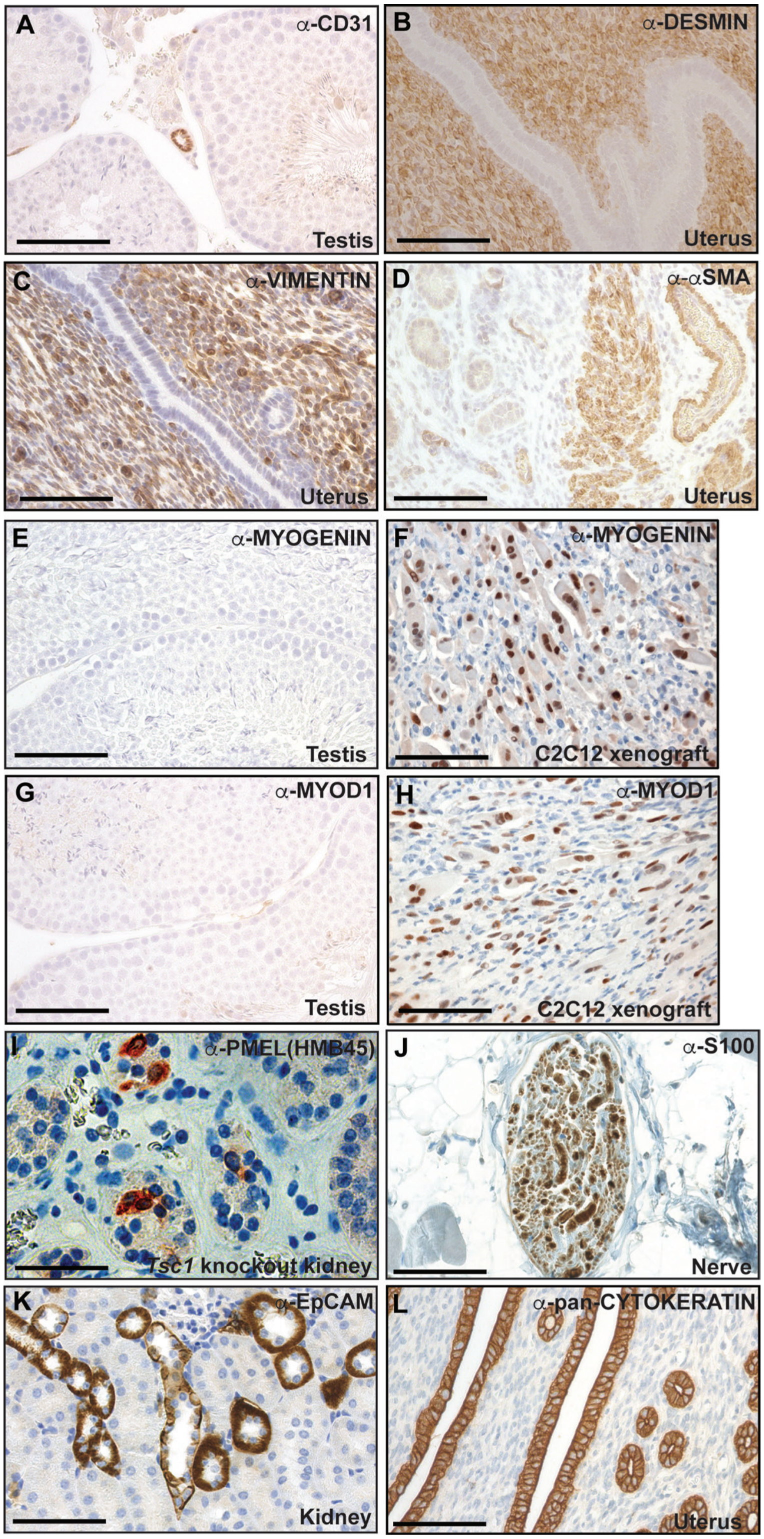

Supplementary Figure 4: Positive and negative immunohistochemical staining controls of antibodies used in this study. (**A**) Mouse testis showing CD31 positivity in a blood vessel and negative staining in tubules. (**B**) Mouse uterus showing DESMIN positivity in the stroma and negative staining in epithelia. (**C**) Mouse uterus showing VIMENTIN positivity in the stroma and negative staining in epithelia. (**D**) Mouse uterus showing alpha-SMA positivity in myometrium and negative staining in stromal and epithelial cells. (**E**) Negative staining for MYOGENIN in testis and (**F**) positive staining in xenograft tumour of the C2C12 myoblast cell line. (**G**) Negative staining for MYOD1 in testis and (**H**) positive staining in xenograft tumour of the C2C12 myoblast cell line. (**I**) Positive staining for PMEL(HMB45) in a *Tsc1* mutant kidney, a mouse model of renal angiomyolipoma (from Gonçalves et al. Nature Communications, 2017, DOI: 10.1038/s41467-017-01514-3). (**J**) Positive staining for S100 in an intramuscular nerve fibre. (**K**) Positive and negative immunoreactivity for EpCAM in renal epithelial tubules. (**L**) Mouse uterus showing positive staining for pan-CYTOKERATIN in epithelia and absence of staining in stroma. Scale bars: 100 µm.

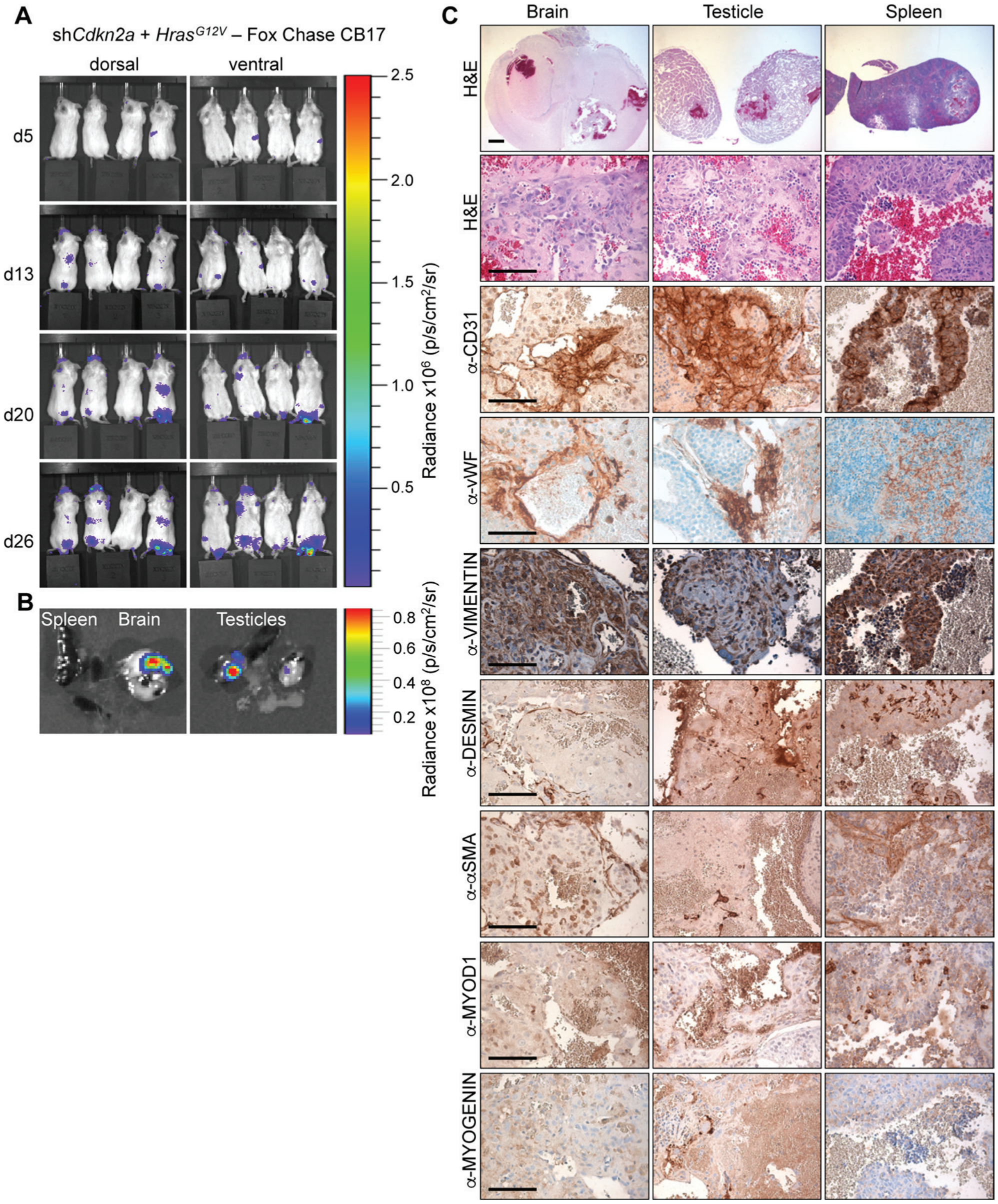

Supplementary Figure 5: ***Hras***^{G12V} **expression plus knockdown of** ***Cdkn2a*** **causes angiosarcomas in Fox Chase CB17 mice.** (**A**) Bioluminescence imaging 5, 13, 20 and 26 days after the injection of MuLE lentiviruses expressing a shRNA against *Cdkn2a* together with *Hras*^{G12V} into the tail vein of 4-6-week-old Fox Chase CB17 mice. (**B**) Bioluminescence imaging showing examples of tumour-bearing organs. (**C**) H&E and immunohistochemical stainings using the indicated antibodies. Low magnification scale bar: 1000 µm and high magnification scale bar: 100 µm.

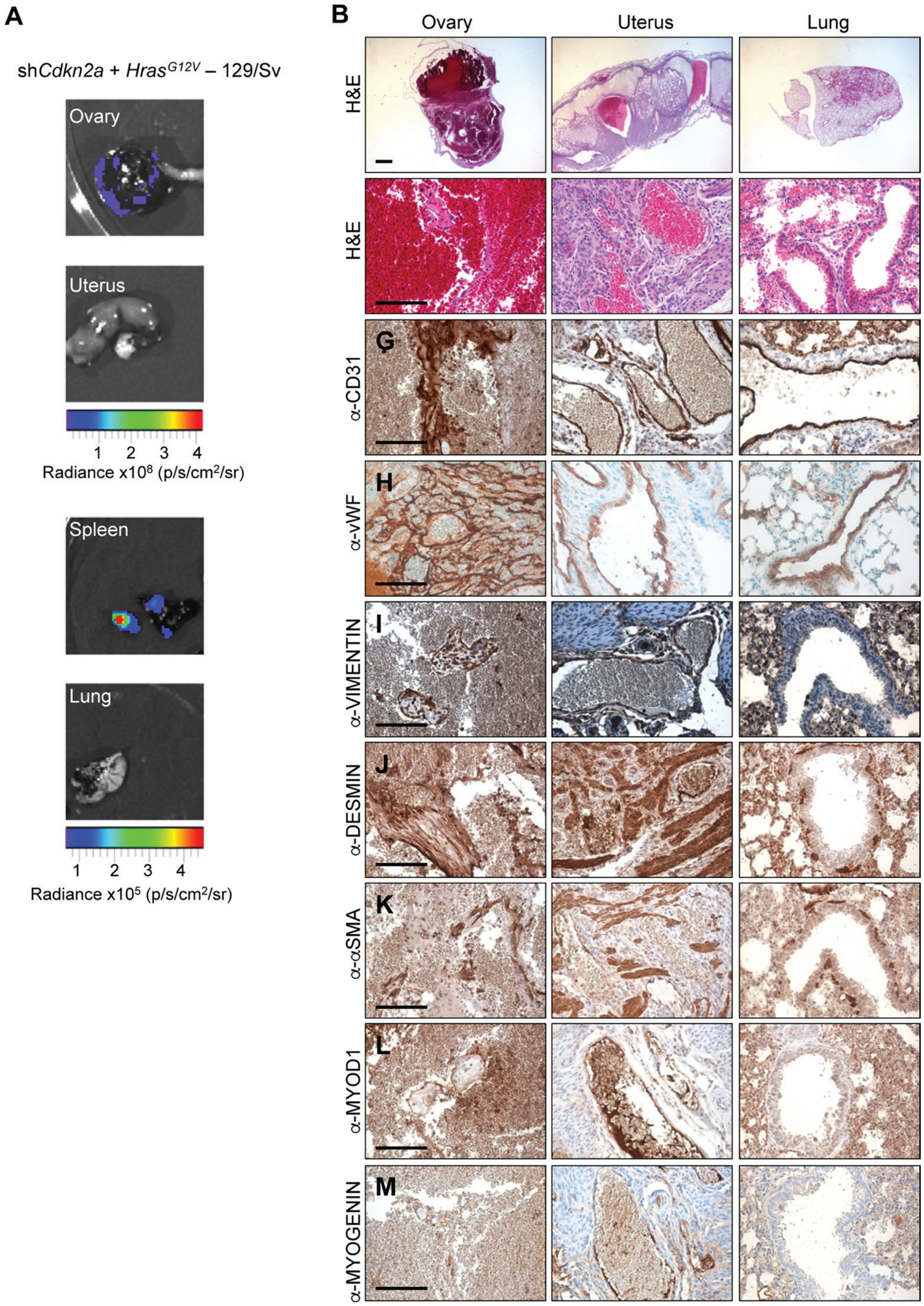

Supplementary Figure 6: Additional information related to Figure 3. (**A**) Bioluminescence imaging showing examples of tumour-bearing organs. (**B**) H&E and immunohistochemical stainings using the indicated antibodies. Low magnification scale bar: 1000 μm and high magnification scale bar: 100 μm.

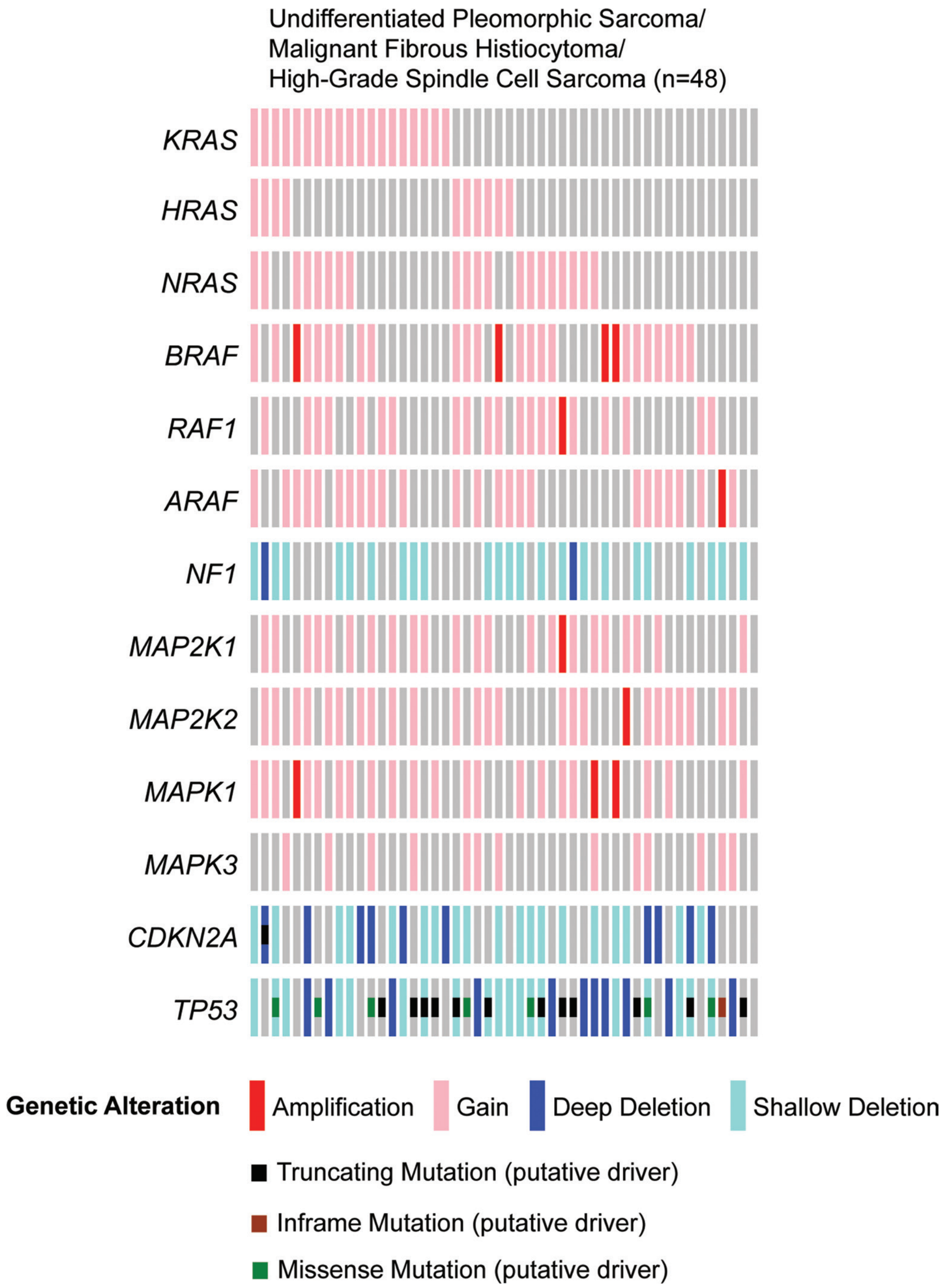

Supplementary Figure 7: Genetic landscape of human UPS tumours. Oncoprint analysis from cBioPortal of the chromosomal copy number status and mutation status of the indicated genes of the RAS-RAF-MEK-MAPK pathway and of *CDKN2A* and *TP53* in 48 cases of human UPS from the TCGA Sarcoma provisional dataset.
